# Detailed deletion mapping of chromosome band 14q32 in human neuroblastoma defines a 1.1-Mb region of common allelic loss

**DOI:** 10.1054/bjoc.2000.1108

**Published:** 2000-06

**Authors:** M Hoshi, N Otagiri, H O Shiwaku, S Asakawa, N Shimizu, Y Kaneko, R Ohi, Y Hayashi, A Horii

**Affiliations:** Departments of Molecular Pathology, Pediatric Surgery, Pediatric Hematology and Oncology, Tohoku University School of Medicine, 2-1 Seiryo-machi, Aoba-ku, Sendai, 980-8575, Japan; Department of Molecular Biology, Keio University School of Medicine, 35 Shinanomachi, Shinjuku, Tokyo, 160-8792, Japan; Department of Cancer Chemotherapy, Saitama Cancer Center, 818 Komuro, Ina, Saitama, 362-0806, Japan

## Abstract

Neuroblastoma (NB) is a well-known malignant disease in infants, but its molecular mechanisms have not yet been fully elucidated. To investigate the genetic contribution of abnormalities on the long arm of chromosome 14 (14q) in NB, we analysed loss of heterozygosity (LOH) in 54 primary NB samples using 12 microsatellite markers on 14q32. Seventeen (31%) of 54 tumours showed LOH at one or more of the markers analysed, and the smallest common region of allelic loss was identified between D14S62 and D14S987. This region was estimated to be 1-cM long from the linkage map. Fluorescence in situ hybridization also confirmed the loss. There was no statistical correlation between LOH and any clinicopathologic features, including age, stage, amplification of *MYCN* and ploidy. We further constructed a contig spanning the lost region using bacterial artificial chromosome and estimated this region to be approximately 1.1-Mb by pulsed-field gel electrophoresis. Our results will contribute to cloning and characterizing the putative tumour-associated gene(s) in 14q32 in NB. © 2000 Cancer Research Campaign


*British Journal of Cancer* (2000) **82**(11), 1801–1807
© 2000 Cancer Research Campaign
DOI: 10.1054/ bjoc.2000.1108, available online at http://www.idealibrary.com on IDEAL®

### Detailed deletion mapping of chromosome band 14q32 in human neuroblastoma defines a 1.1-Mb region of common allelic loss

**M Hoshi[1,2], N Otagiri[1,2], HO Shiwaku[1], S Asakawa[4], N Shimizu[4], Y Kaneko[5], R Ohi[2], Y Hayashi[2,3] and A Horii[1]**

Departments of [1]Molecular Pathology, [2]Pediatric Surgery and [3]Pediatric Hematology and Oncology, Tohoku University School of Medicine, 2-1 Seiryo-machi, Aoba-ku, Sendai, 980-8575, Japan; [4]Department of Molecular Biology, Keio University School of Medicine, 35 Shinanomachi, Shinjuku, Tokyo, 160-8792, Japan; [5]Department of Cancer Chemotherapy, Saitama Cancer Center, 818 Komuro, Ina, Saitama, 362-0806, Japan

**Summary** Neuroblastoma (NB) is a well-known malignant disease in infants, but its molecular mechanisms have not yet been fully elucidated. To investigate the genetic contribution of abnormalities on the long arm of chromosome 14 (14q) in NB, we analysed loss of heterozygosity (LOH) in 54 primary NB samples using 12 microsatellite markers on 14q32. Seventeen (31%) of 54 tumours showed LOH at one or more of the markers analysed, and the smallest common region of allelic loss was identified between D14S62 and D14S987. This region was estimated to be 1-cM long from the linkage map. Fluorescence in situ hybridization also confirmed the loss. There was no statistical correlation between LOH and any clinicopathologic features, including age, stage, amplification of *MYCN* and ploidy. We further constructed a contig spanning the lost region using bacterial artificial chromosome and estimated this region to be approximately 1.1-Mb by pulsed-field gel electrophoresis. Our results will contribute to cloning and characterizing the putative tumour-associated gene(s) in 14q32 in NB. © 2000 Cancer Research Campaign

Neuroblastoma (NB) is one of the most common malignant diseases of childhood. The most frequent genetic abnormality reported to date is the deletion of the short arm of chromosome 1 (Brodeur et al, 1981; Gilbert et al, 1984). Recent investigations have reported that the frequency of loss of heterozygosity (LOH) in 1p ranged from 20 to 89% (Fong et al, 1989, 1992; Suzuki et al, 1989; Weith et al, 1989; Takayama et al, 1992; Caron et al, 1993, 1996; Schleiermacher et al, 1994; Takeda et al, 1994; Martinsson et al, 1995), and two or more tumour suppressor genes have been thought to be located in chromosome 1p (Schleiermacher et al, 1994; Takeda et al, 1994; Caron et al, 1995; Cheng et al, 1995). However, the genes on chromosome 1 responsible for NB have not yet been reported. On the other hand, allelic loss in the long arm of chromosome 14 (Suzuki et al, 1989; Fong et al, 1992; Takayama et al, 1992; Srivatsan et al, 1993; Caron et al, 1996) has also been frequently reported, and these investigations have suggested that a tumour suppressor gene(s) might be located in chromosome bands 14q32-qter (Suzuki et al, 1989; Fong et al, 1992; Takayama et al, 1992). Furthermore, in chromosome arm 14q, commonly deleted regions have also been reported in several other malignancies, including renal cell carcinoma (Kovacs and Frisch, 1989), colorectal carcinoma (Sasaki et al, 1989; Young et al, 1993) and ovarian carcinoma (Bandera et al, 1997). These deleted regions are likely to overlap with that in NB; there may be some common pathways or genes associated with the tumorigenesis of these tumours. As the first step towards the isolation and characterization of the tumour-associated gene responsible for initiation and/or progression of NB, we analysed 54 paired samples of tumour and normal tissues from patients with NB for LOH analyses at 12 microsatellite loci. Herein we report the localization of a putative tumour suppressor gene to a 1.1-Mb region on 14q32.

*Received 14 June 1999*
*Revised 10 January 2000*
*Accepted 17 January 2000*

*Correspondence to:* A Horii

### MATERIALS AND METHODS

### Patients with NB analysed

The Ethical Committee of Tohoku University School of Medicine approved all protocols and samples used in the present study. A total of 54 patients with NB were analysed. Mass screening was performed for 52 of 54 patients, and 36 (69%) were positive. Clinical characteristics of the tumours are summarized in Table 1. The ages of the patients at the time of operation ranged from 6 months to 8 years. They were staged according to Evans' classification (Evans et al, 1971). Primary sites of NB were as follows: 37 from adrenal gland, 12 from retro-peritoneum and five from mediastinum. Six patients had been treated with chemotherapy before operation. None had been treated with radiotherapy.

### Tissue samples

Tumour and corresponding normal tissues were obtained from NB patients treated at the Tohoku University Hospital (Sendai, Japan), Saitama Cancer Center Hospital (Saitama, Japan), and their related institutions within the period from September 1988 to June 1995. Tumour samples were frozen in liquid nitrogen immediately after resection and stored at –80°C until use. In each case, corresponding normal tissue was obtained from one of the following: peripheral

**Table 1** Summary of samples analysed

| Stage | Number of patients[a] Younger[f] | Older[g] | Total | *MYCN* – | + | Chromosome ploidy [b]AP | [c]DP | [d]ND |
|---|---|---|---|---|---|---|---|---|
| I | 9 (2) | 1 (0) | 10 (2) | 9 | 0 | 8 | 1 | 1 |
| II | 14 (4) | 4 (4) | 18 (8) | 18 | 0 | 12 | 2 | 4 |
| III | 5 (1) | 4 (0) | 9 (1) | 9 | 0 | 6 | 0 | 3 |
| IV | 2 (0) | 13 (5) | 15 (5) | 7 | 6 | 5 | 3 | 7 |
| IVs | 2 (1) | 0 (0) | 2 (1) | 2 | 0 | 2 | 0 | 0 |
| Total | 32 (8) | 22 (9) | 54 (17) | 45 | 6 | 33 | 6 | 15 |

[a]Number of patients who showed loss of heterozygosity on chromosome arm 14q are indicated in brackets. [b]AP: aneuploid, [c]DP: diploid, [d]ND: unable to examine. [e]Amplification of *MYCN* was not determined in three patients. [f]Younger: younger than 12 months of age at operation. [g]Older: older than 12 months of age at operation.

**Table 2** Nucleotide sequences of the primers for each microsatellite marker

| Locus symbol | Annealing temperature | Nucleotide sequence CA strand | GT strand |
|---|---|---|---|
| D14S990 | 55°C | AGCCACATGCATCCTCTGTC | GAATAAAGTTGCACTGTGACTG |
| D14S63 | 55°C | CTCTAACACTCACCATGTTCAT | CTGCCAGAGAGCCACACTG |
| D14S81 | 55°C | ACTTATCCTAAAATGAAACTNCA | CAGAGCAGGACCCTTTCTCA |
| D14S265 | 55°C | GTTTTTGGCTATTATGAATATGG | CATACATATGCATGCACTCTG |
| D14S62 | 55°C | GAGGCTTCAGCCTTGCTGT | TCTGTGTGTGATGTATCTGCT |
| D14S987 | 55°C | CTTGAGGCCAGGAGTTTGAG | AGCTGAACTATTTTAATTCAATTGT |
| D14S979 | 55°C | TGTACCACCACCTCCTTATAC | GATGCTCAATGAACAGCCTGA |
| D14S65 | 55°C | CCCTAAAGATCCCTGCCATC | CTGTTCAAATCAGGACCA |
| D14S998 | 55°C | CATCCAAGGGAAAATGAGAG | TTCTCAGATAAATGACAGCGT |
| D14S267 | 55°C | GTTCTTTAAGAGCCAAACATAC | GGATTATTTTNCAAGGTTTCGTA |
| D14S250 | 55°C | TGATGCAGCAATCACTGGAC | ACCCCTGCATTGTTTGAG |
| D14S260 | 55°C | TTTGAAAATGTAAAAGTGTTATTCC | AAAACGGTCACTATGTTTTCCA |

Markers are ordered from the centromeric end to the telomeric one on chromosome band 14q32.

white blood cells, non-cancerous tissue obtained mainly by biopsy of the liver, or non-metastatic lymph node. Some of the samples were obtained from formalin-fixed and paraffin-embedded tissues.

### Analysis of LOH

To obtain the genomic DNAs for microsatellite analysis, we performed micro dissection; tissue samples embedded in paraffin blocks were cut into 50-μm-thick slices and deparaffinized with xylene. The genomic DNAs were extracted as previously described (Kimura et al, 1996). DNAs from fresh frozen tumour tissues and peripheral blood cells were also extracted as previously described (Sambrook et al, 1989; Sato et al, 1990). Based on the Généthon human linkage map (Dib et al, 1996), nucleotide sequences of primer sets for microsatellite markers on chromosome band 14q32 were designed to amplify less than or around 100-bp so that we could perform polymerase chain reaction (PCR) amplification from the DNA prepared from paraffin sections. Nucleotide sequences and annealing temperatures of these primers are summarized in Table 2. In each primer set, the primer corresponding to the DNA strand harbouring the CA dinucleotide repeat was labelled with [$\gamma$-$^{32}$P] ATP, and PCR amplification was performed as described previously (Kimura et al, 1996). We performed electrophoreses of the PCR products in 6% polyacrlamide/8 M urea/32% formamide denatured gels, obtained autoradiograms, and analysed them densitometrically. When a sample exhibited some heterozygosity, allelic imbalance, probably loss, was assigned if the intensity of one of the bands in the tumour sample showed more than a 50% reduction from that of the corresponding normal tissue. At least two independent experiments as well as experiments with the labelled antisense primer (corresponding to the strand for the GT dinucleotide repeat) were performed to confirm our results. Fluorescence in situ hybridization (FISH) analysis was also performed in the critical cases to confirm our microsatellite analysis (see below).

### Isolation of the genomic clones harbouring the commonly deleted region

A yeast artificial chromosome (YAC) clone y964D10 harbouring microsatellite marker D14S62 that had been isolated by PCR-based screening was purchased from Research Genetics (Huntsville, AL, USA). A cosmid library was constructed using the total yeast DNA of this YAC clone by methods described previously (Ariyama et al, 1995), and a clone named c14–16 harbouring D14S62 was isolated by colony hybridization. This clone was used for FISH analysis (see below). BAC clones were obtained by screening two total human BAC libraries; one was constructed by Asakawa et al (1997), and the other was purchased from Research Genetics (Huntsville, AL, USA). From the latter, we prepared a PCR-based BAC screening system and isolated BAC clones. The BAC DNAs were extracted by automated DNA

 *© 2000 Cancer Research Campaign*

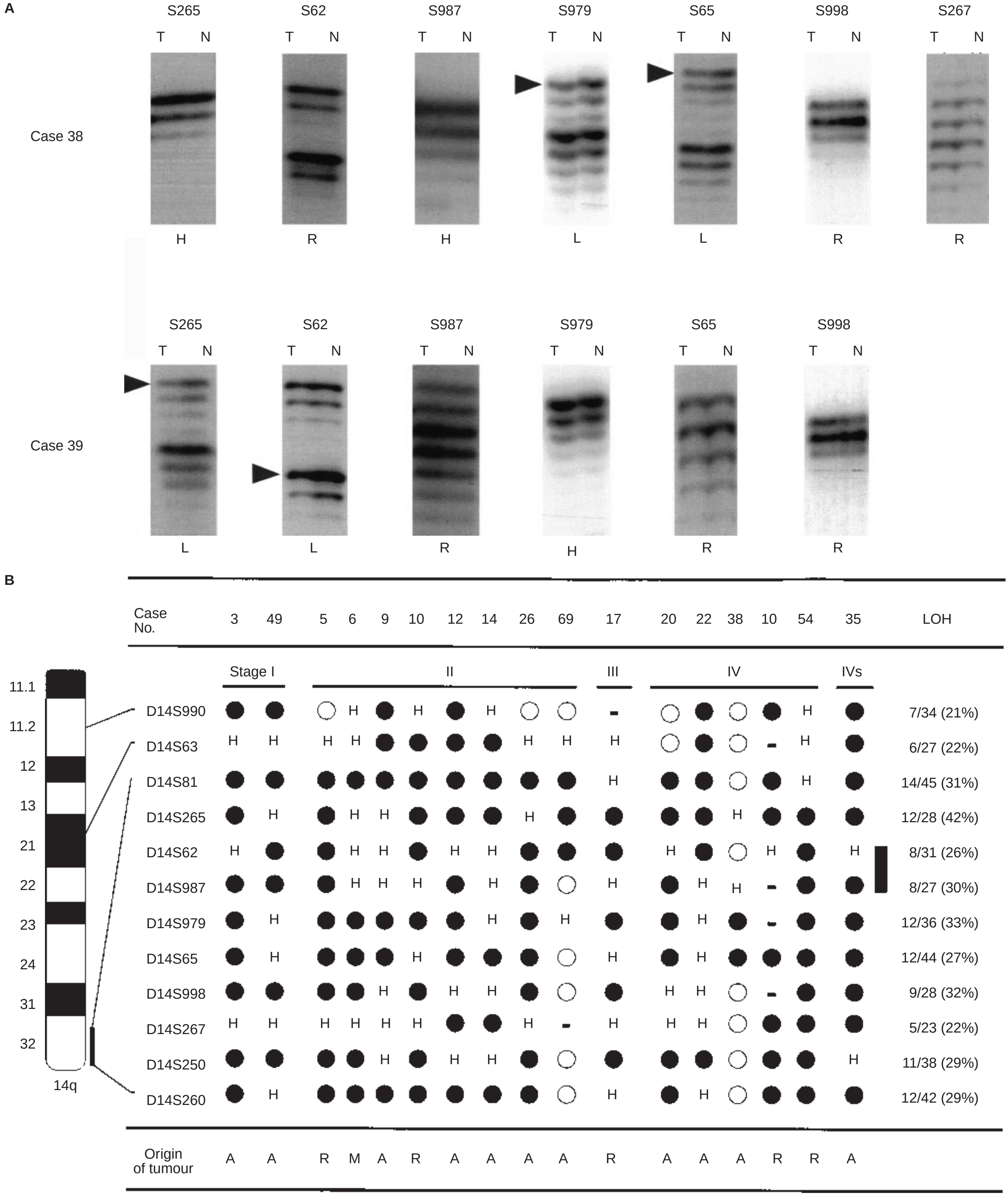

**Figure 1** (**A**) LOH study employing microsatellite markers on chromosome band 14q32. Intensities of bands of DNA from tumours indicated by arrows are significantly reduced when compared with those of corresponding normal tissues. Cut-off value of allelic loss applied in this study was more than 50% reduction. In case 38, the intensities of the upper bands indicated by arrows were reduced by 65% and 60% at D14S979 and D14S65 respectively. In case 69, the intensities of the bands indicated by arrows were reduced by 58% and 56% from the corresponding normal bands at D14S265 and D14S62 respectively. T, tumour samples; N, normal tissue samples; H, homozygous; L, loss of heterozygosity; R, retention. (**B**) Summary of 17 neuroblastoma patients who showed allelic losses on chromosome band 14q32. A closed bar on the right side indicates the commonly deleted region. Open circle, retained heterozygosity; closed circle, loss of heterozygosity; H, constitutionally homozygous; –, not determined. Origins of the tumour are indicated at the bottom; A, adrenal gland; R, retro-peritoneum; M, mediastinum

*© 2000 Cancer Research Campaign*

extract system PI-100 (Kurabo, Neyagawa, Japan), and the DNAs were used for FISH analyses and the contig construction of the commonly deleted region.

### Analysis of FISH

To confirm the results of the microsatellite analysis, we performed dual-colour FISH according to the methods described previously (Nagase et al, 1997). The clones were labelled with either biotin-16-dUTP or digoxigenin-11-dUTP for FISH probes, and the samples from critical cases were hybridized with these labelled probes for at least 2 days at 37°C. Then, to obtain fluorescence signals, they were hybridized with anti-avidin–FITC (fluoroscein isothiocyanate) and anti-digoxigenin–rhodamine, counterstained with DAPI to obtain the FISH images. These images were then incorporated with LEICA DMRXA microscope with COHU CCD black and white camera 4910 and analysed by LEICA QFISH software and Q550CW hardware package (LEICA, Heerbrugg, Switzerland). In the FISH analysis, we analysed allelic losses or imbalances by counting the number of FISH signals in more than 50 nuclei. To confirm the results of FISH, we changed the labelling of the probe in each labelled clone and performed independent experiments with each probe.

### Construction of the BAC contig

We constructed a sequence-ready BAC contig between D14S62 and D14S267, approximately 8-cM region. First we selected BAC clones corresponding to the microsatellite markers between D14S62 and D14S267, including D14S62 and D14S267, using the PCR-based screening system coupled with colony hybridization. Then we determined the nucleotide sequences of both ends of each isolated BAC clone, designed the primer sets for PCR, and performed screening to select the flanking BAC clones. To confirm this sequence-ready BAC contig, we performed Southern hybridization of all the BAC clones with the PCR primers. Consequently we obtained a BAC contig covering the region between D14S62 and D14S267, including the commonly deleted region, as shown in Figure 3. To measure the insert sizes of the six BACs covering the commonly deleted region, we performed *Not*I digestion of DNA prepared in agarose plugs followed by pulsed-field gel electrophoresis (PFGE). Southern blot hybridizations were done with total human DNA labelled with [α-$^{32}$P] dCTP to measure the insert sizes of the BAC clones.

### Analysis of MYCN amplification

DNAs extracted from tumour samples were analysed by slot blot hybridization according to the methods described by Kafatos et al (1997). Hybridization was performed using *MYCN* cDNA as the probe, and the ratio of the signal intensity of each tumour sample to the corresponding normal one was measured densitometrically. We evaluated the tumour specimens as *MYCN* amplification-positive if the tumour showed at least twice the intensity of the normal tissue.

### Analysis of chromosomal ploidy

Chromosomal ploidy of tumour cells was analysed using the G-banding method or flowcytometry. There were 15 patients whose tumour cells showed no division in culture within 24 h; thus we examined 39 informative cases for analysis of chromosomal ploidy.

### RESULTS

Initially, we investigated the clinicopathological features of 54 NB cases in this study. We first investigated the *MYCN* amplification. All but three patients, cases 3, 20 and 22, were examined; amplification of *MYCN* was detected only in six, all of whom were stage IV. We also examined the chromosomal ploidy of the tumour cells;

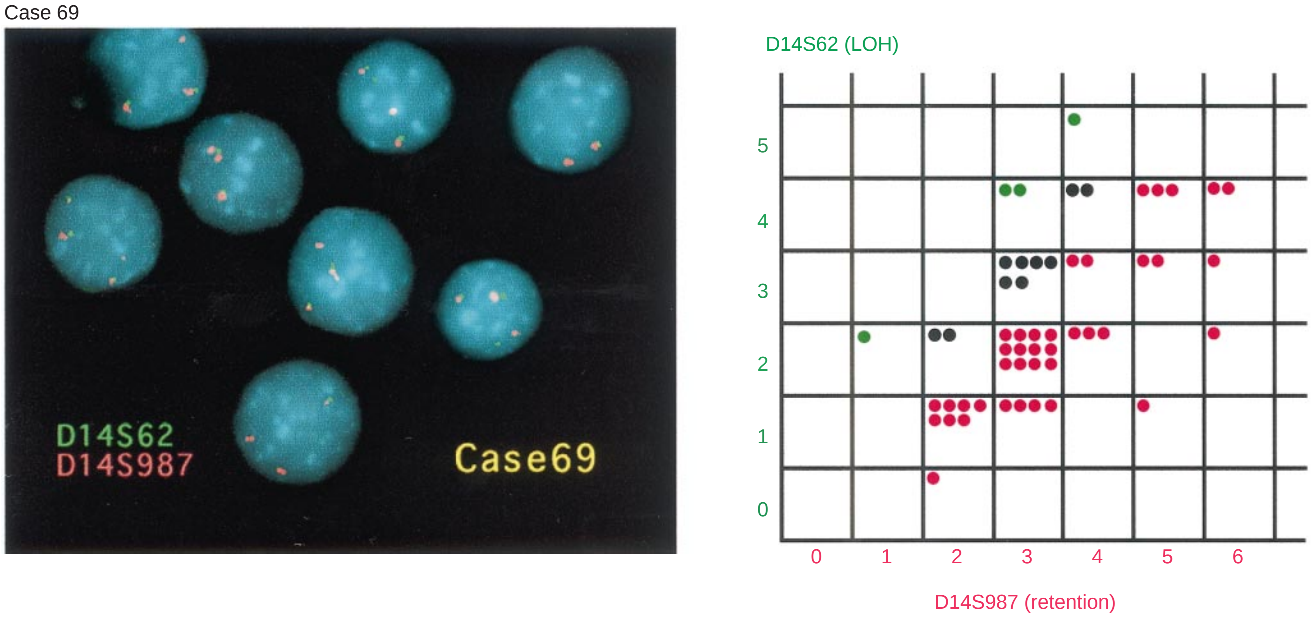

**Figure 2** Dual-colour FISH image of Case 69. Three red signals for b726B5 at D14S987 were observed in this image, whereas only two or fewer green signals for c14–16 at D14S62 were observed in each interphase spread. The counts of FISH signals in case 69 are shown by coloured dots in the right side. In most of the nuclei, the number of signals for D14S987 is greater than that for D14S62, confirming the results of microsatellite analyses

 *© 2000 Cancer Research Campaign*

**Table 3** Summary of cases showing 14q LOH

| Case no. | Age | Stage | N-myc gene amplification | Ploidy | Mass screening |
|---|---|---|---|---|---|
| 3 | 10 months | I | ND | AP | MS |
| 5 | 8 months | II | – | AP | MS |
| 6 | 1 year 1 month | II | – | AP | MS |
| 9 | 1 year 10 months | II | – | AP | MS |
| 10 | 2 years | II | – | AP | FL |
| 12 | 8 months | II | – | AP | MS |
| 14 | 6 months | II | – | ND | MS |
| 17 | 1 year | III | – | AP | FL |
| 20 | 4 year | IVA | ND | ND | FL |
| 22 | 1 year 1 month | IVA | ND | AP | FL |
| 26 | 1 year 11 months | II | – | ND | MS |
| 35 | 6 months | IVs | – | AP | MS |
| 38 | 3 years | IVA | – | DP | FL |
| 40 | 6 years | IVA | + | ND | FL |
| 49 | 8 months | I | – | AP | MS |
| 54 | 1 year 5 months | IVB | – | AP | FL |
| 69 | 8 months | II | – | AP | MS |

AP, aneuploid; DP, diploid; ND, not examined. MS, picked up by mass screening; FL, failed to pick up by mass screening.

33 (85%) of 39 informative cases were aneuploid. These data are summarized in Table 1. We could not detect any significant correlations between age of onset, chromosomal ploidy and amplification of *MYCN*. There were significant correlations between the stage classification (early stages: stage I, II, and IVs; late stages: stage III and IV except for IVs) and age of onset or chromosomal ploidy or amplification of *MYCN* ($P$ = 0.00008, $P$ = 0.04 and $P$ = 0.00006 respectively). Since 69% of our cases were selected by mass screening, the above mentioned correlation may have been influenced.

We then analysed allelic imbalances of chromosome band 14q32 in the 54 cases. We first analysed seven microsatellite loci (D14S81, D14S265, D14S62, D14S65, D14S267, D14S250 and D14S260) by a microsatellite-based PCR-LOH method and then added five more microsatellite loci (D14S990, D14S63, D14S987, D14S979 and D14S998) for a total of 12 loci on chromosome arm 14q. Seventeen (31%) of 54 patients showed allelic imbalances, probably LOHs, at one or more loci on 14q. None of the six patients who had been treated with chemotherapy before their operations showed allelic imbalances. In 15 (88%) of the 17 patients that showed allelic imbalances, such imbalances, predicted to be LOHs, were observed at almost all informative loci; deletion of large segment of chromosome arm 14q including 14q32 was suggested in these patients. On the other hand, cases 38 and 69 had more specific patterns of loss. In case 38, the LOHs were observed at the D14S979 and D14S65 loci; the intensities of the upper bands were reduced by 65% and 60% respectively, from those of the corresponding normal bands. In this patient, heterozygosity was retained at two flanking markers D14S62 and D14S998. In case 69, the intensity of the bands indicated by arrows was reduced by 58% and 56% from the corresponding normal bands at D14S265 and D14S62 loci respectively. Heterozygosity was retained at the D14S987 and D14S65 loci in

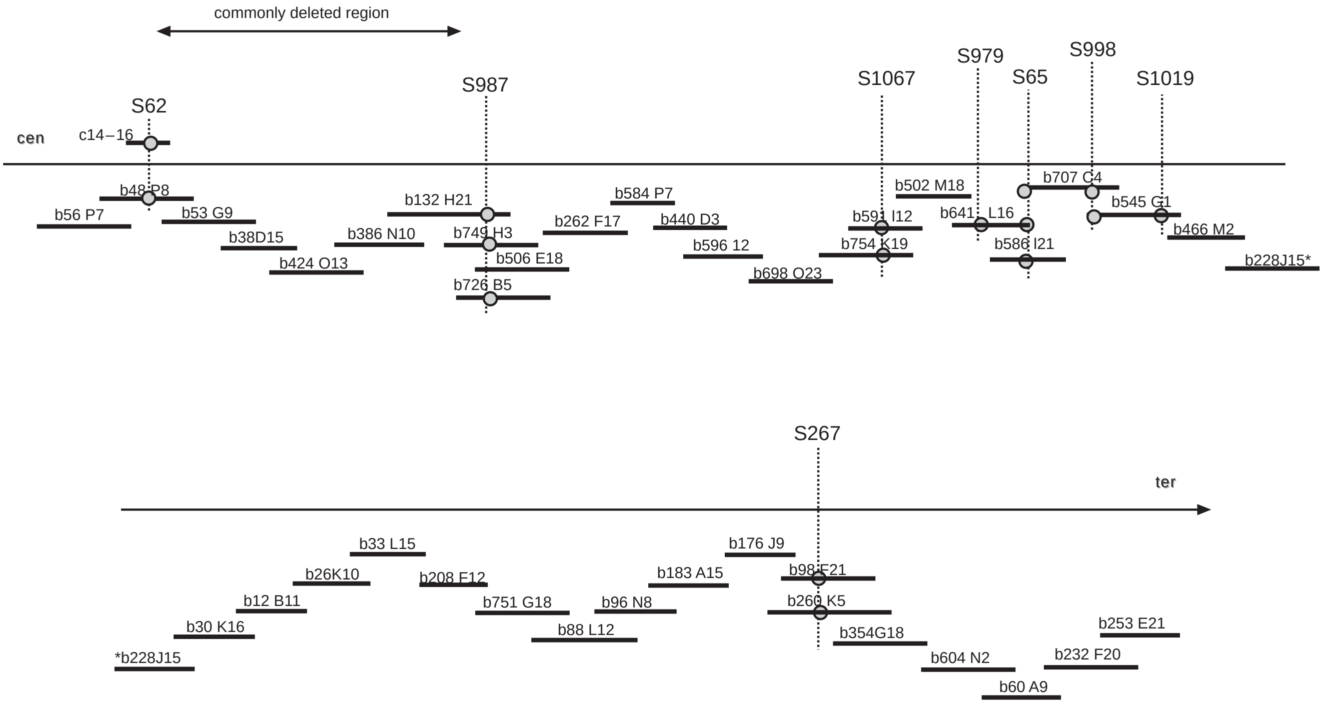

**Figure 3** A sequence-ready BAC contig covering the deleted region. Based on this contig, the commonly deleted region was covered with six overlapping BAC clones, and the physical distance was approximately 1.1 Mb. The centromeric portion is on the upper layer, and telomeric portion is on the bottom layer. The clone b228J15, indicated by an asterisk, bridges the two clones, b466M2 (in the upper layer) and b30K16 (in the bottom layer). The cosmid clone c14–16 that was isolated initially is also shown. Sizes of the BAC clones covering the commonly deleted region are also provided in the parenthesis

© 2000 Cancer Research Campaign this tumour. Thus these two cases showed interstitial deletions on chromosome band 14q32; according to the results, the smallest region of common allelic loss was defined between D14S62 and D14S987. The results of the microsatellite analysis are indicated in Figure 1. None of the tumours showed microsatellite instability (MSI) at any of the tested microsatellite loci.

To confirm the microsatellite analysis on chromosome band 14q32, dual-colour FISH analysis was performed in case 69. We could not sufficiently analyse case 38, because the tumour specimen was not for FISH analysis. Typical results are shown in Figure 2. A majority of the nuclei showed three signals for b726B5, a BAC clone harbouring microsatellite marker D14S987 (rhodamine, red), but only two signals for c14–16, a cosmid clone harbouring D14S62 (FITC, green). Signal numbers that were counted in more than 50 nuclei are shown by dots on the right side of Figure 2. This result indicates a decrease in copy number at D14S62 from that at D14S987. These results were consistent with those of microsatellite analyses in case 69. We further performed FISH analyses with marker sets, D14S65 and D14S998, D14S990 and D14S62, and D14S990 and D14S998; the results showed signal count reductions at D14S65 and D14S62 with no reduction but rather triploidy at both D14S990 and D14S998 respectively (data not shown). These results supported our results of PCR-LOH analysis; case 69 appeared to harbour an interstitial deletion between D14S81 and D14S62. Tumours with LOHs on 14q are summarized in Table 3; case 38 was diploid, and case 69 was aneuploid.

To investigate further, we constructed a BAC contig spanning an 8-cM region including the commonly deleted region, as shown in Figure 3. The relative order of the microsatellite markers that were not determined precisely in the linkage map was found to be as follows: 14cen-D14S62-D14S987-D14S1067-D14S979-D14S65-D14S998-D14S1019-D14S267-14qter. The region between D14S62 and D14S267, approximately 8-cM according to the linkage map, was covered with 32 clones of minimum tiling path, and the commonly deleted region was covered with a minimum tiling path consisting of six overlapping BAC clones. PFGE with *Not*I digested BAC DNA was performed to measure the physical distance of the commonly deleted region; it was estimated to be approximately 1.1-Mb.

### DISCUSSION

In the present study, 31% allelic loss was observed on chromosome arm 14q, in good agreement with other reports on the LOH analysis of 14q (Suzuki et al, 1989; Fong et al, 1992; Takayama et al, 1992). The centromeric and telomeric borders of the commonly deleted region were D14S62 and D14S987; the interval was 1-cM according to the Généthon human linkage map (Dib et al, 1996). We established that the physical size of this region is approximately 1.1-Mb by PFGE. This region overlaps that of the previous work (14q32-qter) by Takayama et al (1992), which was done using restriction fragment length polymorphism (RFLP) markers. Another report on NB (Theobald et al, 1999) suggests the presence of two or more loci for putative tumour suppressor genes on chromosome 14 by RFLP and microsatellite analysis; one of them is between markers D14S1 and D14S16 and the other one is between markers D14S17 and D14S23 in 14q32. However, those areas are large; our study may be a first report of fine deletion mapping of 14q32 in NB.

Recently, disruption of the DNA mismatch repair system has been found to important associations with carcinogenesis of various human cancers and to involve microsatellite instability (MSI). No MSI was observed in this study, in good agreement with those in previous reports (Schleiermacher et al, 1994; White et al, 1995). MSI is thus not generally involved in the tumorigenesis in NB.

In NB, 1p deletion and 17q amplification are associated with the age of onset and/or the clinical stage of NB, or with chromosomal ploidy (Fong et al, 1989; Takeda et al, 1994; Bown et al, 1999). Amplification of *MYCN* and 17q was associated with poor prognosis (Brodeur et al, 1992; Bown et al, 1999) as was a relatively large deletion of chromosome 1p (Schleiermacher et al, 1994; Takeda et al, 1994; Caron et al, 1995). Thus genetic alterations in NB appear to be associated with some clinical features. In this study, however, there was no correlation between LOHs on 14q32 and any clinical feature, including stage, chromosomal ploidy, *MYCN* amplification and age of onset ($P = 0.38$, $P = 0.31$, $P = 0.45$ and $P = 0.19$ respectively, by Fisher's exact test). In the present study, 36 cases were picked up by mass screening of 52 patients; the efficiency was 69% (36/52). More than 80% of the patients examined showed aneuploidy. Although we cannot exclude the possibility of a sampling bias, there is also no correlation between the mass screening and 14q32 LOH ($P = 0.83$). It is interesting that the LOH on 14q in this study showed the same frequency as reported previously despite the possible sampling bias.

In this study, we further performed FISH analysis to confirm the microsatellite analysis. By FISH analysis, we could detect an interstitial allelic deletion in case 69 that was consistent with the microsatellite analysis. In the LOH analysis of this study, we cannot exclude the possibility of 'pseudo' allelic loss derived from aneuploidy, but the FISH analysis clearly demonstrated an allelic imbalance, so we could confirm that the LOHs by microsatellite analysis were caused by true allelic losses in 14q32.

In this study, we further constructed a sequence-ready BAC contig in the region spanning D14S62 through D14S267. A minimum tiling path consisting of 32 overlapping BAC clones covered the entire region. The smallest region of common allelic loss in NB identified in the present study was approximately 1.1-Mb. Further investigations are necessary to understand and characterize the role of a putative tumour suppressor gene in 14q32 in the pathogenesis of NB. Moreover, the contig presented in this study will be a great help in cloning and characterizing the gene(s) that is closely associated with pathogenesis of tumours of the colorectum, ovary, and kidney as well as NB.

### ACKNOWLEDGEMENTS

The authors are grateful to Dr Tasuke Konno (Tohoku University) for providing samples, and to Dr Barbara Lee Smith Pierce (the Life Science Coordinator for the University of Maryland Asian Division) for editorial work in the preparation of this manuscript. This work was supported by the Ministry of Education, Science, Sports, and Culture of Japan.

### REFERENCES

Ariyama T, Inazawa J, Ezaki T, Nakamura Y, Horii A and Abe T (1995) High-resolution cytogenetic mapping of the short arm of chromosome 1 with newly isolated 411 cosmid markers by fluorescence in situ hybridization: the precise order of 18 markers on 1p36.1 on prophase chromosomes and 'stretched' DNAs. *Genomics* **25**: 114–123

Asakawa S, Abe I, Kudoh Y, Kishi N, Wang Y, Kubota R, Kudoh J, Kawasaki K, Minoshima S and Shimizu N (1997) Human BAC library; construction and rapid screening. *Gene* **191**: 69–79

Bandera CA, Takahashi H, Behbakht K, Liu PC, LiVolsi VA, Benjamin I, Morgan MA, King SA, Rubin SC and Boyd J (1997) Deletion mapping of two potential

*© 2000 Cancer Research Campaign*

chromosome 14 tumor suppressor gene loci in ovarian carcinoma. *Cancer Res* **57**: 513–515

Bown N, Cotterill S, Lastowska M, O'Neill S, Pearson AD, Plantaz D, Meddeb M, Danglot G, Brinkschmidt C, Christiansen H, Laureys G and Speleman F (1999) Gain of chromosome arm 17q and adverse outcome in patients with neuroblastoma. *N Engl J Med* **340**: 1954–1961

Brodeur GM, Green AA, Hayes FA, Williams KJ and Tsiatis AA (1981) Cytogenetic features of human neuroblastoma cell lines. *Cancer Res* **41**: 4678–4686

Brodeur GM, Azar C, Brother M, Hiemstra J, Kaufman B, Marshall H, Moley J, Nakagawara A, Saylors R, Scavarda N, Schneider S, Wasson J, White P, Seeger R, Look T and Castleberry R (1992) Neuroblastoma, effect of genetic factors on prognosis and treatment. *Cancer* 70 (suppl.): 1685–1694

Caron H, van Sluis P, van Hoeve M, de Kraker J, Bras J, Slater R, Mannens M, Voûte PA, Westerveld A and Versteeg R (1993) Allelic loss of chromosome 1p36 in neuroblastoma is of preferential maternal origin and correlates with N-*myc* amplification. *Nat Genet* **4**: 187–190

Caron H, Peter M, van Sluis P, Speleman F, de Kraker J, Laureys G, Michon J, Brugiéres L, Voûte PA, Westerveld A, Slater R, Delattre O and Versteeg R (1995) Evidence for two tumor suppressor loci on chromosomal bands 1p35–36 involved in neuroblastoma: One probably imprinted, another associated with N-*myc* amplification. *Hum Mol Genet* **4**: 535–539

Caron H, van Sluis P, de Kraker J, Bökkerin J, Egeler M, Laureys G, Slater R, Westerveld A, Voûte PA and Versteeg R (1996) Allelic loss of chromosome 1p as a predictor of unfavorable outcome in patients with neuroblastoma. *N Engl J Med* **334**: 225–230

Cheng NC, van Roy N, Chan A, Beitsma M, Westerveld A, Speleman F and Versteeg R (1995) Deletion mapping in neuroblastoma cell lines suggests two distinct tumor suppressor genes in the 1p35–36 region, only one of which is associated with N-*myc* amplification. *Oncogene* **10**: 291–297

Cox DW (1994) Report of the first international workshop on human chromosome 14 mapping 1993. *Cytogenet Cell Genet* **66**: 2–9

Cox DW, Billingsley G and van TT Nguyen (1994) A linkage map of human chromosome 14, including 13 gene loci. *Genomics* **23**: 331–337

Dib C, Faure S, Fizames C, Samson D, Drouot N, Vignal A, Millasseau P, Marc S, Hazan J, Seboun E, Lathrop M, Gyapay G, Morissette J and Weissenbach J (1996) A comprehensive genetic map of the human genome based on 5264 microsatellites. *Nature* **380**: 152–154

Evans AE, D'Angio GJ and Randolph J (1971) A proposed staging for children with neuroblastoma: children's cancer study group A. *Cancer* **27**: 374–378

Fong CT, Dracopoli NC, White PS, Merrill PT, Griffith RC, Housman DE and Brodeur GM (1989) Loss of heterozygosity for the short arm of chromosome 1 in human neuroblastomas: correlation with N-*myc* amplification. *Proc Natl Acad Sci USA* **86**: 3753–3757

Fong CT, White PS, Peterson K, Sapienza C, Cavenee WK, Kern SE, Vogelstein B, Cantor AB, Look AT and Brodeur GM (1992) Loss of heterozygosity for chromosomes 1 or 14 defines subsets of advanced neuroblastoma. *Cancer Res* **52**: 1780–1785

Gilbert F, Feder M, Balaban G, Brangman D, Lurie DK, Podolsky R, Rinaldt V, Vinikoor N and Weisband J (1984) Human neuroblastomas and abnormalities of chromosomes 1 and 17. *Cancer Res* **44**: 5444–5449

Gyapay G, Morissette J, Vignal A, Dib C, Fizames C, Millasseau P, Marc S, Bernardi G, Lathrop M and Weissenbach J (1994) The 1993–94 Genethon human genetic linkage map. *Nat Genet* **7**: 246–339

Hofker MH, Smith S, Nakamura Y, Teshima I, White R and Cox DW (1990) Physical mapping of probes within 14q32, a subtelomeric region showing a high recombination frequency. *Genomics* **6**: 33–38

Kafatos FC, Jones CW and Efstratiadis A (1979) Determination of nucleic acid sequence homologies and relative concentrations by a dot hybridization procedure. *Nucleic Acids Res* **7**: 1541–1552

Kimura M, Abe T, Sunamura M, Matsuno S and Horii A (1996) Detailed deletion mapping on chromosome arm 12q in human pancreatic adenocarcinoma: Identification of a 1-cM region of common allelic loss. *Genes Chromosomes Cancer* **17**: 88–93

Kovacs G and Frisch S (1989) Clonal chromosome abnormalities in tumor cells from patients with sporadic renal cell carcinomas. *Cancer Res* **49**: 651–659

Laureys G, Speleman F, Versteeg R, van der Drift P, Chan A, Leroy J, Francke U, Opdenakker G and van Roy N (1995) Constitutional translocation t (1;17) (p36.31–p36.13; q11.2–q12.1) in a neuroblastoma patient. Establishment of somatic cell hybrids and identification of PND/A12M2 on chromosome 1 and NF1/SCYA7 on chromosome 17 as breakpoint flanking single copy markers. *Oncogene* **10**: 1087–1093.

Martinsson T, Weith A, Cziepluch C and Schwab M (1989) Chromosome 1 deletions in human neuroblastomas: generation and fine mapping of microclones from the distal 1p region. *Genes Chromosomes Cancer* **1**: 67–78

Martinsson T, Sjöberg R-M, Hedborg F and Kogner P (1995) Deletion of chromosome 1p loci and microsatellite instability in neuroblastomas analyzed with short-tandem repeat polymorphisms. *Cancer Res* **55**: 5681–5686

Nagase S, Yamakawa H, Sato S, Yajima A and Horii A (1997) Identification of a 790-kilobase region of common allelic loss in chromosome 10q25–q26 in human endometrial cancer. *Cancer Res* **57**: 1630–1633

Sambrook J, Fritsch EF and Maniatis T (eds) (1989) *Molecular Cloning: A Laboratory Manual*, 2nd edn. Cold Spring Harbor Laboratory Press, Cold Spring Harbor 1989

Sasaki M, Okamoto M, Sato C, Sugio K, Soejima J, Iwama T, Ikeuchi T, Tonomura A, Miyaki M and Sasazuki T (1989) Loss of constitutional heterozygosity in colorectal tumors from patients with familial polyposis coli and those with nonpolyposis colorectal carcinoma. *Cancer Res* **49**: 4402–4406

Sato T, Tanigami A, Yamakawa K, Akiyama F, Kasumi F, Sakamoto G and Nakamura Y (1990) Allelotype of breast cancer: cumulative allele losses promote tumor progression in primary breast cancer. *Cancer Res* **50**: 7184–7189

Schleiermacher G, Peter M, Michon J, Hugot JP, Vielh P, Zucker JM, Magdelenat H, Thomas G and Delattre O (1994) Two distinct deleted regions on the short arm of chromosome 1 in neuroblastoma. *Genes Chromosomes Cancer* **10**: 275–281

Shizuya H, Birren B, Kim U-J, Mancino V, Slepak T, Tachiiri Y and Simon M (1992) Cloning and stable maintenance of 300-kilobase-pair fragments of human DNA in *Escherichia coli* using an F-factor-based vector. *Proc Natl Acad Sci USA* **89**: 8794–8797

Srivatsan ES, Ying KL and Seeger RC (1993) Deletion of chromosome 11 and 14q sequences in neuroblastoma. *Genes Chromosomes Cancer* **7**: 32–37

Suzuki T, Yokota J, Mugishima H, Okabe I, Ookuni M, Sugimura T and Terada M (1989) Frequent loss of heterozygosity on chromosome 14q in neuroblastoma. *Cancer Res* **49**: 1095–1098

Takayama H, Suzuki T, Mugishima H, Fujisawa T, Ookuni M, Schwab M, Gehring M, Nakamura Y, Sugimura T, Terada M and Yokota J (1992) Deletion mapping of chromosomes 14q and 1p in human neuroblastoma. *Oncogene* **7**: 1185–1189

Takeda O, Homma C, Maseki N, Sakurai M, Kanda N, Schwab M, Nakamura Y and Kaneko Y (1994) There may be two tumor suppressor genes on chromosome arm 1p closely associated with biologically distinct subtypes of neuroblastoma. *Genes Chromosomes Cancer* **10**: 30–39

Theobald M, Christiansen H, Schmidt A, Melekian B, Wolkewitz N, Christiansen NM, Brinkschmidt C, Berthold F and Lampert F (1999) Sublocalization of putative tumor suppressor gene loci on chromosome arm 14q in neuroblastoma. *Genes Chromosomes Cancer* **26**: 40–46

Weith A, Martinsson T, Cziepluch C, Brüderlein S, Amler LC, Berthold F and Schwab M (1989) Neuroblastoma consensus deletion maps to 1p36.1–2. *Genes Chromosomes Cancer* **1**: 159–166

White PS, Maris JM, Beltinger C, Sulman E, Marshall HN, Fujimori M, Kaufman BA, Biegel JA, Allen C, Hilliard C, Valentine MB, Look T, Enomoto H, Sakiyama S and Brodeur GM (1995) A region of consistent deletion in neuroblastoma maps within human chromosome 1p36.2–36.3. *Proc Natl Acad Sci USA* **92**: 5520–5524

Young J, Leggett B, Ward M, Thomas L, Buttenshaw R, Searle J and Chenevix-Trench G (1993) Frequent loss of heterozygosity on chromosome 14 occurs in advanced colorectal carcinomas. *Oncogene* **8**: 671–675

*© 2000 Cancer Research Campaign*
